# Mr. Jeffreys' Cases in Surgery, from the Records of the St. George's and St. James's Dispensary

**Published:** 1822-12-01

**Authors:** 


*Cases in Surgery, selected from the Records of the Author's Practice at the St. George's and St. James's Dispensary; and illustrating the Nature and Mode of Treatment of Strumous or Scrofulous Ophthalmia; the sedative Powers of Tartar Emetic in the Cure of Local Inflammations, when administered internally; the Treatment of the Mammary, or Milk Abscess; and the beneficial Effects of Elm Bark as a cheap Substitute for Sarsaparilla. With Two Plates.* By Henry Jeffreys, Esq. Senior Surgeon to the St. George's and St. James's Dispensary; Assistant Surgeon to the Lock Hospital; and formerly a Surgeon in the Third Regiment of Foot Guards. Octavo, pp. 237. London, 1820.

The St. George's and St. James's Dispensary has now existed about five years, and has distributed extensive medical relief to the poor of those and the neighbouring parishes. We are sorry to learn, from the publication before us, that the funds of this very useful institution are rather on the decline—a state, we believe, which is participated by most of the dispensaries in this metropolis. There is no good without its attendant evils in this world. The rapid increase of these charitable institutions throughout the great and small towns of this country, has become rather a burthen on the subscribers; and the public begin to perceive, that the majority of them are instituted from private motives, and for selfish purposes, though there can be no doubt that, even so, the good effects are the same, as far as the suffering poor are concerned. We have reason to believe, also, that, with the really necessitous, there is now an afflux of patients to these dispensaries, whose circumstances might fairly enable them to employ the humble and junior classes of general practitioners—who accordingly suffer materially, not only in a pecuniary point of view, but in the circumscription of their proper field of observation and experience. Thus, while the *few* medical officers attached to these institutions, shall profit by the extensive field of practice laid open to them, the *many* young men just entering on their professional career, will be absolutely cut off from all sources of observation, excepting among the better classes, very few of whom will employ them at this early period of their lives. Here is a very serious evil: and it is extremely problematical if even the medical officers themselves derive so much advantage, pecuniary or practical, as is generally supposed. In a pecuniary point of view, we have the very best means of knowing that these institutions have fallen far short, indeed, of their expected influence in leading to private practice; and when we contemplate the contrast between the constitutions, habits, accommodations, &c. of the poor and the affluent, we must be convinced that the medical practice in these two classes is widely different. Still, with all these draw-backs, the institutions in question have opened excellent sources of observation for their medical officers, and the profession at large has been greatly benefited, from time to time, by the results of these observations.

The volume before us makes very humble pretensions, aspiring no farther than to convey some plain practical information to the junior classes of the profession, on subjects which but rarely come under the student's observation during his attendance as a pupil at public hospitals. This is the kind of information, by the way, which is most useful; though both young and old will hunt after rare cases and extraordinary operations, which they may never again meet with in the course of an extensive practice.

The first paper in this work is a long one, on the nature and treatment of scrophulous ophthalmia, and contains many judicious observations. It is not necessary for us to dwell on it here in very minute detail, but merely exhibit a brief sketch of its contents. Mr. J. first mentions tinea ciliaris and lippitudo, two affections which are frequent attendants on strumous ophthalmia, though often independent of that disease. The *first* of these shows itself in the form of small pimples or pustules along the roots of the eye-lashes, which break and exude a puriform fluid that glues the edges of the lids together, often, in aggravated cases, occasioning the eye-lashes to drop out, and sometimes destroying them altogether, leaving small ulcers, and a morbidly sensible state of the eye. In *lippitudo* the cartilaginous edges of the eye-lids are red and excoriated, and it depends on a morbid condition of the meibomian glands, the secretion from which is morbidly increased and glutinous. This and tinea ciliaris are frequently mistaken for each other, and, indeed, they are often conjoined.

The treatment of both these complaints consists of alterative and tonic medicines, occasional mercurial purgatives, mild but nourishing diet, pure and dry atmosphere. These last are easily recommended, but how are they to be obtained by the poor of London, to whom the complaints in question are almost entirely confined? The topical applications are solutions of sulph. zinci, cupri, &c. while the edges of the lids should be touched at bed time with the ung. hyd. nitrat. or some such ointment.

Of strumous ophthalmia the tunica conjunctiva is the principal seat. Mr. J. divides it into acute and chronic. It seldom attacks infants at the breast; but from the time they are weaned, "it is perhaps the most common disorder of the eye to which young children are subject, and very often presents the first indication of a strumous diathesis." When neglected it becomes a frightful source of imperfect vision by the specks and opacities which it leaves on the cornea. In the acute form the great impatience of light is one of the peculiar characteristics of the scrophulous ophthalmia.

"Around the circumference of the cornea an effusion of serum occasionally takes place from the inflamed vessels, elevating the conjunctiva into a circular vesication about half a line or more in breadth, frequently occupying the entire margin of the cornea, and exhibiting a peculiar reddish brown appearance. Along with this symptom, there may often be seen clusters of minute vesicles, or pustules, occupying the same line, or scattered separately over the cornea and conjunctiva. These pustules vary in size according to the part of the conjunctiva on which they appear, being commonly smallest upon the cornea, and increasing as they approach towards the angle where that membrane is reflected over the internal superficies of the lids; and may be considered as a distinguishing symptom of this disease. In mild cases they contain a serous fluid: but where the inflammation is of a more active character, they become filled with coagulable lymph, or albumen; and, in still severer cases, with pus." 16.

If the inflammatory action runs high, or is not checked by proper means, the pustules are apt to break, and form ulcers, which constitute the most formidable symptom of this complaint. The pain is greatly increased, owing to the friction made upon the ulcers, the intolerance of light becomes excessive, and the inflammation is often augmented, in which case the ulcer may spread, either by the formation and exfoliation of successive sloughs, and, if not checked, may penetrate the substance of the cornea, and lay the anterior chamber open, which event is followed by the discharge of the aqueous humour, and protrusion of the iris.

When the phlogosis is milder, instead of the ulcer spreading by slough, lymph becomes effused on its surface, and a halo of transparent appearance is formed round the margin of the ulcer, which is a favourable circumstance, and should not be interfered with.

When the acute form does not terminate in resolution or speedy healing of the ulcers, it becomes converted into the chronic state—a form the most usually met with, and differing from the acute chiefly in duration, and the absence of active inflammation, the vessels of the conjunctiva remaining in a state of passive congestion, and gorged with red blood. There is, however, the same distressing intolerantia lucis, profuse lachrymation, ulcers covered with effused lymph, and remaining in an indolent state.

In the treatment of strumous ophthalmia, the first indication, of course, is to relieve the local disease—and that must be done by a mild antiphlogistic course. Local blood-letting he did not always find necessary, and he thinks general bleeding can hardly ever be required. "Where the inflammatory action runs higher than ordinary, or where it has been suddenly and violently augmented by the formation of ulcers in the cornea; or where a rapid and extensive effusion of lymph is taking place, the increased impetus of the vessels, should be moderated by the application of leeches to the under eye-lids." It will seldom, he observes, be necessary to repeat this operation. Emetics and purgatives, darkness, and regulated diet, are, of course, powerful items in the methodus medendi—especially as in most children, among the lower classes, who labour under this disease, we observe tumid abdomens, and disordered abdominal secretions. Here the exhibition of an emetic, (a measure too much neglected in modern times,) followed by a purgative, will be of great benefit. "The best purge is calomel combined with scammony, jalap, or rhubarb, administered at intervals of two or three days." In recent attacks of the acute form, the inflammation, pain, and irritation may be moderated by gently astringent evaporating lotions, applied either warm or cold, according to the feelings, ("or prejudices," Mr. J. says,) of the patient. We have found no application more grateful to inflamed and irritable eyes than decoction of poppies, with a considerable quantity of extract of conium dissolved in it—say half an ounce of the extract in a quart of fomentation. This should be applied warm five or six times a day.

Mr. Jeffreys's further observations on the general treatment of the acute and chronic forms, contain only common and well-known directions. In respect to certain parts of the local management, he observes that the pustules which form in this disease, should never be opened by art, as they are then more liable to degenerate into ulcers—the most formidable and distressing symptom of the disease. The severity of the pain should be relieved by leeches to the under eye-lid, assisted by a brisk mercurial purge, and the eye kept wet and secured from light. When the ulcer has pierced the cornea, and the iris lies protruded, the ulcer should be touched every other day with a bit of argenti nitras scraped to a point; or a solution of the same (two or three grains to an ounce of water) may be injected over the eye twice or thrice a day. If the prolapsed portion of iris remain protruded beyond the level of the conjunctiva, it must be destroyed by caustic, or snipped off with scissars.

The second paper in the work before us is on the sedative powers of tartar emetic in local inflammations. Both Dr. Balfour and Mr. Jeffreys have underrated the extent to which this medicine has been used in medical and surgical practice by their brethren at large. For the last thirty years, to our knowledge, there is scarcely a form of inflammation, or indeed of febrile excitement, in which all ranks of the profession have not been in the habit of prescribing antimonials, and generally the tartrite, as a controller of vascular action. Mr. Jeffreys, we imagine, is not justified in saying that, "of late years, it (tartar emetic) has seldom been directed but as an emetic or diaphoretic." Now we are quite confident, that, in nine cases out of ten, where it is prescribed by modern practitioners, it is with the view of *lessening the activity of the circulation*, whether that be by increasing the cutaneous and various other secretions, by causing nausea at the stomach, or by any direct sedative effect which it may have on the circulating organs.

Our author states, that when he first read Dr. Balfour's work, he could not bring himself to give full evidence to all his assertions respecting the powers of tartar emetic; but, on putting the medicine to the test of experience at the dispensary, "the result far surpassed any expectations that he could have formed of it, and has been sufficient to satisfy his mind that its sedative powers are such, as not only to reduce very considerably the necessity of general and local blood-letting, in a great variety of topical inflammations, but, in many, to supersede it altogether." This we believe, and therefore, at charitable institutions, where economy is a cardinal virtue, and where the palates of patients are not quite so delicate as their purses, the medicine in question may prove a valuable succedaneum for leeches and other modes of sanguineous depletion. The diseases in which our author has administered the medicine were strictly surgical, and the mode of its exhibition was that recommended by Dr. Balfour, viz. two, three, or four grains dissolved, with an ounce of Epsom salts, in six or eight ounces of water, of which mixture the patient is to take two or three table spoonfuls every half hour or oftener, until vomiting is excited; after which, the dose is to be repeated at intervals of three, four, or six hours, according to circumstances.

"Exhibited in the manner I have described, this medicine appears to exercise a very powerful influence over the arterial system, restraining its action, and diminishing its vigour in a manner, and with a rapidity, that is possessed by few other remedies, that I am acquainted with. In many cases the velocity and the strength of the pulse yield to it in a few hours; the tumefaction, pain, and inflammation of the part affected subside, almost, as by a miracle; the febrile excitement, thirst, and restlessness, which accompany and mark the symptomatic fever arising from local inflammation, are relieved and disappear with the same rapidity. And in none of the cases in which I have directed it, and among them have been some delicate and weakly women, have I met with any complaints of its violent action, nor any objections to its continued use. In other instances these effects are more slow in taking place; but in none, in which I have tried it, has it failed to moderate the inflammatory action, and very considerably to reduce the necessity of general and local blood-letting." 96.

A great number of cases of local inflammation treated by tartar emetic are related in illustration of the foregoing remarks, for which we must refer to the work itself.

The third paper contains a needlessly minute journal of a fatal case of stricture and ulceration of the œsophagus, together with the appearances on dissection. The patient was 61 years of age, and during the eight preceding years, had been subject to cough and expectoration of phlegm each winter, which symptoms disappeared in the summer. About six weeks before the date of application, the patient began to feel an uneasiness in his throat, and a difficulty in swallowing his food, which gradually increased, accompanied with pain in the stomach, and emaciation. Besides the difficulty of deglutition, there was also a sensation of much uneasiness about the lower part of the œsophagus, with soreness and distension of stomach, which was generally relieved by large eructations. There was constriction about the root of the tongue, and pain stretching up the throat into the ears and behind the angles of the lower jaw, aggravated by blowing the nose or sneezing. Bougies, plain and armed, were long tried, and with little or no success—indeed we cannot help thinking that the caustic bougies did more harm than good, although they sometimes appeared to lessen the irritability of the parts at the time. He was under treatment from the 14th May, till the 5th November, 1818, when he terminated a wretched and most painful state of existence.

We think that, under such desperate circumstances as the patient was placed in, and where the stricture was clearly proved to be just opposite the cricoid cartilage, an operation might have been perfectly justifiable. If a man can live for years with a canula in the trachea, why should he not be capable of living with one in the œsophagus? As for cutting down upon the œsophagus and opening into that passage, the dexterity of a Cooper, and of many other surgeons, would easily accomplish such a task, with little risk of life to the patient.

*Dissection.* The body was exceedingly emaciated. The œsophagus being slit open, presented the following appearances.

"At that part which lies immediately behind the cricoid cartilage, the area of the canal was nearly obliterated, by a morbid thickening and induration of its coats, for the space of a quarter of an inch or more. The internal membrane was here much thicker and firmer than usual, and, for the space of an inch above and below the contraction, it was ulcerated throughout its whole superficies. The surface of the ulcer was covered with a soft, flocculent kind of slough, of a pale straw colour; and its edges were jagged, abrupt, and irregular. The glands at the root of the tongue were very large and prominent. The epiglottis appeared to be larger, and the glottis to gape wider than usual. Below the ulcer the internal membrane of the œsophagus was redder and more vascular than ordinary,* but did not exhibit any other morbid appearance. The stomach was exceedingly contracted and quite empty; its villous coat was thrown into numerous rugæ; it had a reddish brown colour, and was smeared over with a quantity of yellowish green viscid mucus. Both the larger and smaller intestines were contracted into fleshy chords not thicker than the middle finger. The smaller intestines presented no morbid appearance, and contained nothing but bile and mucus." 147.

The mucous membrane of the colon was of a dark colour, but the rest of the viscera were healthy.

We have had a considerable number of cases of this kind under our care, but none of them proved fatal during our attendance. We kept a drain on the surface opposite the strictured part, and persevered with the common bougie twice a week, or seldomer if the irritability was great.

The fourth essay on the treatment of mammary or milk abscess contains nothing new or particular. Mr. Jeffreys thinks that when the abscess bursts and discharges the slough or core, the breast is too much neglected by practitioners in general, and the consequence is, that indurations and inequalities of the breast remain, which prove a source of alarm, if not actual mischief, afterwards.

"I think the objects in view may be better and more expediti-

---

"* This appearance went off after the parts had been in maceration previous to their being put into spirits."

ously obtained by a mode of proceeding which I have long made use of, and which is very simple and easy, being merely the application of stripes of linen, or calico, spread with equal parts of the soap and adhesive plasters, in such a manner as to envelope and support the whole breast. Each stripe of this plaster should be nearly an inch and a half wide, and long enough to reach from a little below the clavicle to two inches below the breast. They should be applied from below upwards, beginning alternately on the outer and inner circumferences of the breast, and so continued till the whole of it be enveloped. Each succeeding stripe should cover the upper half of the one upon which it is placed, and should be so applied as to make a uniform but moderate pressure upon the whole breast. If there be but one ulcer, or opening, the application of the plasters may be so managed, that the last one or two shall be placed over the ulcer. By this means it will not be necessary to renew the whole of the plasters at each dressing; it being sufficient, in general, merely to remove those covering the sore, in order to give vent to any accumulated matter, and to clean the ulcer. The surface of the sore may be covered with a bit of lint; and, if the granulations should be exuberant, they may be kept down by a solution of the nitrate of silver, or the camphorated vitriolic lotion of Bates." 158.

The paper succeeding the above, contains a case of ill-conditioned ulcer on the ala nasi, of three years standing, successfully treated. Ulcers on the nose resembling lupus and noli me tangere are not very unfrequently met with, and sometimes prove very baffling under the best modes of treatment.

Sarah Chambers, 18 years of age, of scrophulous diathesis, came under our author's care on the 22d March, 1819. She stated, that three years previously an angry pimple on the ala nasi spread, in a few days, into a painful, irritable, and superficial ulcer, which never exceeded the size of a shilling, yet never had healed, though she had been under several eminent surgeons. During the preceding summer she had been at Margate, where, in addition to the nasal sore, she became affected with ulceration in the throat, and painful and difficult deglutition, which still continued, sometimes better and sometimes worse. Although her general health did not seem much deteriorated, she had lost flesh, had a muddy complexion, irregular bowels and catamenia. An issue had been made in her arm a month before, since which, she thought the ulcer less irritable. At the time of admission the ulcer was larger than a six-pence, nearly circular in shape, its surface slightly elevated above the surrounding integuments, and covered with large, smooth, shining granulations, its edges bordered with thin red cuticle. The tonsils were enlarged and prominent, with an ulcer on the left one, covered with a thick white slough. She had a furred tongue, and a full, hard abdomen. She was ordered rhubarb and the blue pill to regulate the bowels, and a pint of the decoctum ulmi compositum, (hereafter to be noticed,) to drink daily. By the 6th April, the ulcer on the tonsil was healed, but that on the nose was rather worse. An eighth of a grain of the oxymuriate of mercury was given night and morning, and a lotion applied to the sore, composed of opium, conium, and sulphate of zinc. Under a pretty long course of this, her health improved, her bowels were rendered regular and natural, and the nasal ulcer completely healed. We cannot but suspect that, in this case, there was a syphilitic taint in the system.

A case of ulcer, of particular character, on the tongue, is next detailed by our author. The kind of ulcer, in question, is not very frequently met with. It is situated in the middle and towards the posterior part of the tongue, commencing in the form of a pimple, which soon becomes a deep, irregular, fissured ulceration, with hard elevated edges, and surrounded, for half an inch, or more, by an induration of the muscular substance of the organ. It takes place in constitutions somewhat out of order, and appears to yield more readily to mercury than to any other remedy. When the ulcer heals, the loss of substance is not restored, and a hardened depression remains.

*Case.* Thomas Williams, 40 years of age, came under our author's care on the 25th November, 1817, for an ulcer on his tongue, of an irregular shape, deep, and fissured. Its surface was covered with a whitish, thick, adhesive matter, the edges being elevated, hard, and irregular, and surrounded by a circumscribed induration of the lingual substance. The ulcer was a little sore during the day; but, towards night, became dry and painful. His voice was somewhat impaired and hoarse, and he complained of slight pain and huskiness in the throat, but without ulcer or inflammation there. His bowels were confined, and he looked much out of health. He stated, that he had been suffering, for some years, from occasional attacks of hæmoptysis and pectoral complaints; but that his present ailment had arisen about three weeks before, in the form of a pimple which soon went into ulceration. He had had a venereal complaint about ten years ago; but was now a married man with several children.

"He was desired to take a pint of the Compound Decoction of Sarsaparilla, daily; to keep the bowels regular, by means of pills, consisting each of three grains of the Pilul. Hydrargyri, and three of Ext. Colocynth. Co. and to touch the ulcer twice or three times a day, with the Linimentum Æruginis. This application occasioned a good deal of pain in the ulcer, shooting into the throat, and behind the ears." 191.

At the end of ten days, a part of the slough had separated from the posterior part of the ulcer, but anteriorly, the sore appeared disposed to spread. His health, however, was improving. The ulcer was now touched once a day with a weak solution of nitrate of silver. Towards the end of December, the remainder of the slough had separated, but the ulcer continued deep and fissured, without disposition to granulate. The decoctum sarsæ was left off, and the patient was put on a course of the liquor arsenicalis. This medicine excited such pain and sickness at stomach, with faintness, cold clammy sweats, &c. that it was necessary to discontinue it. The patient was now directed to take an eighth of a grain, night and morning, of the oxymuriate of mercury, and to continue the caustic solution.

"In a few days the surrounding induration began to disperse; he had less pain in the ulcer, and it put on a more favourable appearance; but did not granulate. When he had taken the pills about fourteen days, his mouth and gums became affected; he had an increased flow of saliva, and complained of shortness of breath and cough. On this account the pills were omitted for a few days, till these symptoms went off, when they were resumed, but at bedtime only. Towards the end of January new cuticle began to descend the sides of the ulcer; and on the 3d of February its surface was entirely skinned over, the surrounding induration was nearly gone, and he was free from pain. Nevertheless, a deep indurated hollow remained where the ulcer had been; and his voice did not perfectly recover its natural tone and power." 192.

He was discharged cured on the 19th of February, and has remained well ever since.

The volume concludes with the narration of several cases illustrating the beneficial effects of elm bark, as a cheap substitute for sarsaparilla. The high price of the latter medicine renders it, of course, inaccessible to the poor; and few dispensaries can afford to exhibit it, to any extent, to the applicants for relief. A cheap substitute, therefore, is a desideratum, and further experience must decide on the pretensions of the elm bark. Our author, indeed, does not rank this homely production with the costly foreign article, but he avers that, prepared as he directs, it is possessed of more medicinal virtues than have generally been attributed to it. The good effects of elm bark in cutaneous diseases have been extolled by several writers, particularly the late Dr. Lettsom, and Dr. Daniel Lysons, of the Gloucester Infirmary. Mr.

Jeffreys has given it an extensive trial at the dispensary, and speaks highly of its virtues. The following is the formula.

" DECOCTUM ULMI COMPOSITUM.

"℞. Decocti Ulmi (P. L.) ferventis oc. viij;

Sassafras Radicis Concisæ,

Guaiaci Ligni Rasi, sing. ℥j;

Mezer. Rad. Corticis ʒiij;

Glycyrrhizæ Rad. Contusæ ℥j.

Decoque per horam, sepone, et cola.

" This decoction, when properly prepared, and strained off, is of a clear brown colour, not unpleasant in its taste, and contains a considerable proportion of amylacious and mucilaginous matter. Administered in the quantity of a pint a day, it appears to increase the insensible perspiration, to restore the appetite, improve the tone and powers of the digestive organs, to strengthen and invigorate the general system, and to cheer and compose the animal spirits. Like the decoctions of the woods in general, its action may be said to be alterative and tonic; and its use may be persisted in for a considerable length of time, without overloading and oppressing the stomach, or producing any other unpleasant symptom. Its action upon the bowels has, in general, a tendency to produce constipation rather than otherwise." 198.

Our author has administered this decoction, both alone and in conjunction with other medicines, as antimonials, liquor potassæ, liquor arsenicalis, oxymuriate of mercury, the mineral acids, &c. in a considerable number of cases. The complaints in which he has found it most serviceable, were those which very frequently follow venereal ulcers on the genitals, when mercury has been improperly or inefficiently exhibited —as nodes, and painful tumefactions of the periosteum and ligaments, ozœna, cutaneous defœdations, foul and untractable ulcers, scrofulous abscesses, chronic rheumatism, morbid enlargements and induration of the testicles, &c. For the numerous cases in illustration we must refer to the work itself.

Mr. Jeffreys' volume has not been ushered into the world with any pretension or claim beyond that of recording those facts which daily meet the eye of the surgeon in dispensary practice, and which form the most useful species of knowledge for the young practitioner. We wish every medical officer of a public institution would follow Mr. Jeffreys' example.

